# The Transdiagnostic Role of Emotion Regulation Difficulties and Repetitive Negative Thinking in Depression, Anxiety, and Their Comorbidity

**DOI:** 10.1155/da/9949037

**Published:** 2026-05-04

**Authors:** Eva Herzog, Sebastian Wolf, Thomas Studnitz, Anna Katharina Frei, Britta Seiffer, Jana Welkerling, Johanna-Marie Zeibig, Gorden Sudeck, Celina L. Müller, Mia Maria Günak, Tristan T. Nakagawa, Thomas Ehring, Leonie Sundmacher, Stefan Peters, Anna Lena Flagmeier, Lena Zwanzleitner, Ander Ramos-Murguialday, Keisuke Takano, Barbara Cludius

**Affiliations:** ^1^ Department of Psychology, Clinical Psychology and Psychological Treatment, Ludwig-Maximilians-Universität München, Munich, Germany, uni-muenchen.de; ^2^ Institute for General Practice and Interprofessional Care, University Hospital Tübingen, Tübingen, Germany, uni-tuebingen.de; ^3^ Department of Education and Health Research, Faculty of Economics and Social Sciences, Institute of Sports Science, University of Tübingen, Tübingen, Germany, uni-tuebingen.de; ^4^ Department of Psychology, Clinical Psychology and Psychotherapy, Julius-Maximilians-Universität Würzburg, Würzburg, Germany, uni-wuerzburg.de; ^5^ German Center for Mental Health (DZPG), Munich Site, Munich, Germany; ^6^ Chair of Health Economics, Technical University Munich (TUM), Munich, Germany, tum.de; ^7^ German Association for Health-Enhancing Physical Activity and Exercise Therapy (German: DVGS), Hürth-Efferen, Germany; ^8^ Department of Human Sciences, Institute of Sport Science, Bundeswehr University Munich, Neubiberg, Germany; ^9^ AOK Baden-Württemberg, Stuttgart, Germany; ^10^ Techniker Krankenkasse, Hamburg, Germany; ^11^ Medical Faculty, Institute of Medical Psychology and Behavioral Neurobiology, University of Tübingen, Tübingen, Germany, uni-tuebingen.de; ^12^ Department of Neurology and Stroke, University of Tübingen, Tübingen, Germany, uni-tuebingen.de; ^13^ Tecnalia, Basque Research and Technology Alliance, San Sebastián, Spain; ^14^ Athenea Neuroclinics, San Sebastián, Spain; ^15^ Human Informatics and Interaction Research Institute, The National Institute of Advanced Industrial Science and Technology (AIST), Tsukuba, Japan, aist.go.jp; ^16^ Department of Psychology, Clinical Psychology and Psychotherapy of Adulthood, University of Bremen, Bremen, Germany, uni-bremen.de

## Abstract

This study investigated emotion regulation (ER) difficulties and repetitive negative thinking (RNT) in individuals with depression, anxiety disorders, or both, compared to nonclinical controls. We aimed to determine which aspects of ER difficulties and RNT are transdiagnostic or specific to one of the disorders, and whether the presence of comorbidities is associated with greater ER difficulties and higher levels of RNT. A total of *N* = 731 participants, including *n* = 354 individuals with depression, anxiety disorders, or both (mixed group), and *n* = 377 nonclinical controls, completed the Difficulties in Emotion Regulation Scale (DERS) and the Perseverative Thinking Questionnaire (PTQ). Parametric and nonparametric analyses were conducted to assess group differences and comorbidity effects. The depression and anxiety disorders groups exhibited significantly greater ER difficulties and higher levels of RNT than nonclinical controls. The mixed group showed more pronounced difficulties in certain aspects of ER (nonacceptance of emotional responses, difficulties engaging in goal‐directed behavior, impulse control difficulties, and limited access to ER strategies) and higher levels of RNT compared to the single‐diagnosis groups. No significant differences were found in emotional awareness and clarity across clinical groups. Evidence regarding the relationship between the number of comorbid diagnoses and the severity of the difficulties was less clear. This suggests that specific disorders may have a more significant impact than comorbidity alone. Our findings highlight the transdiagnostic role of ER difficulties and RNT in depression and anxiety disorders and suggest that they may be a promising target for transdiagnostic interventions. Future research should further explore the nuanced ways in which ER difficulties and RNT vary across different mental disorders.

## 1. Introduction

High comorbidity rates among mental disorders have led to the assumption that they share transdiagnostic risk and maintaining processes [1]. Difficulties in ER have been proposed as an important transdiagnostic process [2], and research indicates that ER difficulties are indeed associated with various mental disorders [3]. However, recent findings from a subclinical sample suggest that only certain facets of ER difficulties are transdiagnostic, while others may only be associated with specific disorders [4]. Systematic research linking specific aspects of ER difficulties to certain disorders within clinical samples is currently lacking. In addition, it remains to be investigated whether the differences within clinical groups are attributable to specific diagnoses per se or rather to patterns of comorbidity between these diagnoses.

Comorbidity, defined as the simultaneous presence of two or more mental disorders in an individual [5], is highly prevalent and affects approximately half of all individuals with mental health problems [6–8]. Individuals with depression or anxiety disorders have particularly high rates of comorbid disorders, which even exceed 50% [9, 10]. Depression stands out as one of the most common comorbid disorders for anxiety disorders, and vice versa [11–13]. Consequently, researchers have developed an interest in elucidating the reasons behind the frequent co‐occurrence of these disorders. Are there, perhaps, transdiagnostic processes common to different disorders that serve as risk factors for several mental health conditions?

ER has been proposed as a transdiagnostic risk factor involved in anxiety and depression [2]. Gratz and Roemer [14] proposed a model of ER in the context of psychopathology, defining broad ER abilities that influence how individuals cope with emotional distress. In this model, the authors conceptualize ER as a multidimensional construct “involving the (a) awareness and understanding of emotions, (b) acceptance of emotions, (c) ability to control impulsive behaviors and behave in accordance with desired goals when experiencing negative emotions, and (d) ability to use situationally appropriate ER strategies flexibly to modulate emotional responses as desired in order to meet individual goals and situational demands” [14, p. 42]. Based on this definition, the Difficulties in Emotion Regulation Scale (DERS) was developed [14], comprising six dimensions of difficulties in ER derived through factor analysis. These include (a) lack of awareness of emotional responses, (b) lack of clarity of emotional responses, (c) nonacceptance of emotional responses, (d) limited access to ER strategies perceived as effective, (e) difficulties controlling impulses when experiencing negative emotions, and (f) difficulties engaging in goal‐directed behaviors when experiencing negative emotions.

Existing research using the DERS provides substantial evidence indicating that difficulties in ER are associated with numerous mental disorders, including anxiety disorders and depression [15–19]. Despite the general association of ER difficulties with various mental disorders, it has been suggested that specific difficulties or patterns in ER may be disorder‐specific and therefore unique to each disorder. For instance, Lukas et al. [20] demonstrated a general increase in ER difficulties across a range of mental disorders (e.g., depression and anxiety disorders) compared to the general population, while also showing significant differences between diagnostic groups. Specifically, individuals with depression showed particularly pronounced difficulties compared to other diagnostic categories, especially in domains related to understanding, accepting, and managing emotional experiences. A study by Im and Kahler [21] examined ER difficulties as a potential transdiagnostic mechanism across several disorders (depression, generalized anxiety disorder, social anxiety disorder, and panic disorder). However, their structural equation models did not provide robust evidence for ER difficulties as a transdiagnostic factor applicable to all disorders under study. As the authors note, this may reflect the use of a nonclinical undergraduate sample with lower symptom severity, as well as challenges in modeling ER as a unitary construct despite its well‐established multidimensional structure. These considerations suggest that nonsignificant findings at the global level do not rule out the possibility that specific ER facets may operate transdiagnostically, particularly in clinical samples.

To reconcile these conflicting findings, Shukla and Pandey [4] proposed that only some ER difficulties may function as transdiagnostic processes, while others are disorder‐specific. They empirically tested this assumption by examining the six different dimensions of difficulties in ER according to the DERS, using two subclinical groups: one characterized by anxiety symptoms only and another showing symptoms of both anxiety and depression. In addition, a third group consisted of participants without any anxiety or depressive symptoms. Results indicated that both subclinical groups showed a higher total score on ER difficulties compared to the group without anxiety or depressive symptoms, with the group showing both anxiety and depression symptoms exhibiting the highest level of difficulties in the ER. When analyzing the six specific dimensions of ER difficulties individually, different findings emerged for the two subclinical groups. Nonacceptance of emotional responses, limited access to ER strategies, and impulse control difficulties were found to be uniquely higher in the mixed anxious–depressed subgroup. Conversely, lack of emotional awareness and emotional clarity as well as difficulties engaging in goal‐directed behavior were equally present in both subclinical groups [4]. These results suggest that certain ER difficulties may be specific to particular disorders, though replication in a clinical sample is warranted. Furthermore, these findings do not conclusively indicate whether specific ER difficulties are exclusively related to depression (as opposed to anxiety disorders) or if they are primarily associated with individuals with multiple comorbid mental disorders.

Thus, to expand upon the findings from Shukla and Pandey’s [4] subclinical study and to investigate whether specific ER difficulties are specifically associated with depression or anxiety or whether they arise solely due to the comorbidity between the two disorders, we recruited several participant groups. In addition to a nonclinical control group, we recruited individuals with (a) depression, (b) an anxiety‐related disorder, and (c) comorbid depression and an anxiety‐related disorder, noting that additional comorbid diagnoses were present in some individuals across all three groups. To delve deeper into the relationship between specific ER difficulties and comorbidity, we not only compared the distinct groups but also explored the severity of these difficulties based on the number of disorders or comorbidities, irrespective of the specific type of disorder. This approach allowed for a more nuanced understanding of whether certain ER difficulties are inherently tied to the presence of a particular disorder or whether they manifest in a cumulative manner with an increasing number of comorbidities. This comprehensive investigation aimed to shed light on the intricate interplay between ER difficulties, specific mental disorders, and the co‐occurrence of these disorders in clinical populations.

Existing research consistently indicates that difficulties in ER are associated with specific strategies that hinder effective ER [3, 22]. Among these strategies, RNT has emerged as a focal point of study, with substantial evidence supporting its transdiagnostic relevance through longitudinal and experimental investigations (e.g., [3, 23–25]). Although RNT is widely recognized as a transdiagnostic cognitive process, direct empirical evidence linking specific dimensions of ER difficulties, as operationalized by the DERS, to RNT remains limited. However, several lines of research examining conceptually related constructs provide empirical evidence relevant to understanding links between ER difficulties and RNT, albeit with mixed results. In particular, reduced emotional awareness and clarity, often captured by the broader construct of alexithymia, have been examined in relation to rumination, a key variant of RNT. Some studies report significant associations between rumination and alexithymia across those subdimensions conceptually related to emotional clarity and emotional awareness (e.g., [26]). However, other work suggests that these associations are not uniform across subdimensions of alexithymia and may be partially accounted for by depressive symptoms [27], indicating that links between clarity‐ and awareness‐related difficulties and rumination may depend on contextual factors. Moreover, rumination does not appear to be consistently elevated in individuals with high alexithymia relative to other maladaptive regulatory strategies (e.g., [28]). Beyond awareness‐ and clarity‐related processes, ER‐related constructs conceptually aligned with nonacceptance have also been implicated in RNT. In particular, beliefs regarding the controllability or changeability of emotional responses, closely related to nonacceptance of emotional responses, have been associated with greater engagement in perseverative cognitive strategies, including rumination [29]. At the same time, meta‐analytic evidence indicates that associations between such beliefs and rumination are small on average and highly heterogeneous, cautioning against assumptions of a robust or uniform relationship [30]. In addition, process‐oriented research discusses rumination as potentially linked to avoidance‐oriented cognitive processing, a perspective that is conceptually compatible with DERS dimensions involving nonacceptance of emotional experiences, although avoidance did not consistently mediate the relationship between rumination and psychological symptoms in the tested models [31]. From this perspective, RNT can be understood as a maladaptive regulation strategy that is conceptually compatible with ER difficulties, particularly those involving nonacceptance and limited access to adaptive strategies.

Taken together, the existing literature suggests that links between ER‐related constructs and RNT are heterogeneous, vary across subdimensions and populations, and may depend on contextual factors such as comorbidity. These mixed findings underscore the value of examining RNT not only as a general transdiagnostic process but also alongside specific facets of ER difficulties within clinically diagnosed samples. Moreover, there is compelling evidence that levels of RNT increase with a higher number of comorbid diagnoses [25].

In light of these theoretical convergences and empirical findings, our study aimed to further investigate RNT as a transdiagnostic cognitive strategy relevant in the context of ER difficulties by examining its levels across diagnostic groups and assessing how its occurrence varies with the number of comorbid diagnoses. By scrutinizing the role of RNT in different diagnostic contexts and its relationship with comorbidity, we sought to contribute to a more nuanced understanding of how particular ER strategies may be implicated across diverse mental health conditions and their combinations.

Based on the ER difficulties model, we expected that individuals with a mental disorder would show higher ER difficulties than nonclinical controls.

1. We expected that, compared to nonclinical controls, individuals with an anxiety‐related disorder (i.e., a diagnosis of agoraphobia and/or panic disorder and/or post‐traumatic stress disorder [PTSD]) would show
  a. Greater ER difficulties for each dimension as assessed by the DERS.
  b. Higher levels of RNT as assessed by the Perseverative Thinking Questionnaire (PTQ).
2. Likewise, we expected that, compared to nonclinical controls, individuals diagnosed with depression would show
  a. Greater ER difficulties for each dimension as assessed by the DERS.
  b. Higher levels of RNT as assessed by the PTQ.

Additionally, we expected that the presence of comorbidities would exacerbate ER difficulties and RNT.

1. We expected that individuals with comorbid anxiety‐related disorders and depression (mixed group) would display
  a. Higher scores across all DERS subscales and
  b. Increased levels of RNT (PTQ)

In comparison to the nonclinical control group and the depression‐only and anxiety‐only groups. We also explored the difference between the anxiety‐only and depression‐only groups.

1. Furthermore, we predicted a significant increase as the number of diagnoses increased across groups (0, 1, 2, 3, or more diagnoses) for
  a. The severity of ER difficulties (DERS), as well as
  b. The level of RNT (PTQ).

## 2. Method

### 2.1. Participants

Participants in the clinical groups were selected from the ImPuls study investigating the impact of a group exercise intervention on transdiagnostic and disorder‐specific symptom severities [32]. Recruitment primarily occurred via referrals from inpatient psychiatric departments, family practices, general practitioners, and psychiatric and psychotherapeutic outpatient units between February 2021 and June 2022. Inclusion criteria for the clinical groups included a diagnosis of at least one of the following disorders based on the International Classification of Diseases, 10th Revision (ICD‐10; [33]): major depressive disorders of at least moderate severity, nonorganic insomnia, panic disorder, agoraphobia, or PTSD. Additionally, participants needed to be affiliated with one of the two participating health insurance providers. Exclusion criteria included current mental or behavioral disorders related to psychotropic substances, acute eating disorders, acute bipolar disorder, acute schizophrenia, and acute suicidal ideation or behavior.

Individuals in the nonclinical control group were matched with the full clinical sample in terms of age, gender, and educational status and were recruited between November 2022 and September 2024. Recruitment of nonclinical participants occurred through various channels, including flyers, mailings, word of mouth, and social media (Facebook and Instagram), as well as within the context of other studies. Inclusion criteria for the control group included the absence of the following diagnoses at the time of study participation: bipolar affective disorder (including currently remitted), current depressive episode, remitted recurrent depressive disorder, dysthymia, agoraphobia, social anxiety disorder, panic disorder, generalized anxiety disorder, obsessive–compulsive disorder, posttraumatic stress disorder, somatic symptom disorder, eating disorders, nonorganic insomnia, schizophrenia, schizotypal and delusional disorders, currently remitted schizophrenia, persistent delusional disorder, schizoaffective disorder, mental and behavioral disorders due to psychotropic substances, or hyperkinetic disorder.

For both groups, inclusion criteria additionally required individuals to be between 18 and 65 years old, fluent in German, and not engaged in exercise of at least moderate intensity more than once per week for at least 30 min each time, over a period of 6 weeks during the 3 months prior to study enrollment^1^.

A total of 354 individuals in the clinical group were included in this study: *n* = 223 with a depressive disorder, *n* = 65 with an anxiety‐related disorder (agoraphobia [*n* = 20] and/or panic disorder [*n* = 22] and/or PTSD [*n* = 28]), and *n* = 66 with comorbid depressive and anxiety‐related disorders (agoraphobia [*n* = 17] and/or panic disorder [*n* = 24] and/or PTSD [*n* = 44]). A total of 378 individuals were recruited as a nonclinical control group. One individual was excluded from the analyses for consistently responding with the most extreme response option on every item. The nonclinical control group thus comprised *n* = 377 individuals. The demographic and clinical characteristics of the sample are outlined in Tables 1 and 2.

**Table 1:** Demographic characteristics and comparison of the subgroups.

Characteristic	Total sample (*N* = 731)	Nonclinical control group (*n* = 377)	Depression (*n* = 223)	Anxiety disorders (*n* = 65)	Depression and anxiety disorders (*n* = 66)	Univariate between‐subjects tests for subgroups		
	*n* (%)	*n* (%)	*n* (%)	*n* (%)	*n* (%)	χ^2^ (df)	*p*	Sign. diff in post‐hoc test comparison^l^
Gender^a^	—	—	—	—	—	28.02 (6)	<0.001	CG ≠ D CG ≠ A CG ≠ D&A D ≠ A D ≠ D&A
Women	524 (71.68)	274 (72.68)	148 (66.37)	48 (73.85)	54 (81.82)	—	—	
Men	198 (27.01)	103 (27.32)	72 (32.29)	13 (20.00)	10 (15.15)	—	—	
Other	9 (1.23)	0 (0.00)	3 (1.35)	4 (6.15)	2 (3.03)	—	—	
Employment^b^	—	—	—	—	—	47.07 (6)	<0.001	CG ≠ D CG ≠ A CG ≠ D&A
Unemployed	184 (25.27)	62 (16.45)	71 (32.13)	24 (37.50)	27 (40.90)	—	—	
Employed	479 (65.80)	288 (76.39)	129 (58.37)	34 (53.13)	28 (42.42)	—	—	
Other	65 (8.93)	27 (7.16)	21 (9.50)	6 (9.38)	11 (16.67)	—	—	
Relationship status^b^	—	—	—	—	—	5.65 (3)	0.13	—
Single	311 (42.54)	155 (41.11)	105 (47.09)	30 (64.15)	21 (31.81)	—	—	—
In a relationship	420 (57.46)	222 (69.82)	118 (52.91)	35 (53.85)	45 (68.18)	—	—	—
Years of education^b,c^		—	—	—	—	6.33 (6)	0.39	—
≤10 Years	163 (22.30)	79 (20.95)	45 (20.18)	20 (30.77)	19 (28.79)	—	—	—
11–13 Years	298 (40.77)	159 (42.18)	88 (39.46)	25 (38.46)	26 (39.40)	—	—	—
>13 Years	270 (36.94)	139 (36.38)	90 (40.36)	20 (30.77)	21 (31.82)	—	—	—
	*M* (*SD*)	*M* (*SD*)	*M* (*SD*)	*M* (*SD*)	*M* (*SD*)	—	—	—
Age (years)	41.05 (13.53)^d^	40.89 (13.86)	41.86 (13.29)^e^	39.50 (12.76)	40.76 (13.24)	1.52 (3)^f^	0.678	—
Physical activity (minutes/week), self‐report (BSA‐F)	465.96 (760.32)^g^	540.88 (839.37)^h^	322.71 (493.97)^i^	500.89 (817.65)^j^	463.72 (881.55)^k^	23.99 (3)^f^	<0.001	CG > D D < A

Abbreviations: A, Anxiety disorders; BSA‐F, Physical activity, exercise, and sport questionnaire; CG, Nonclinical control group; D, Depression; D&A, Depression and anxiety disorders.

^a^Seven participants in the nonclinical control group indicated a different gender during screening compared to a later questionnaire. However, through email correspondence and personal interviews, the correct gender was confirmed in all cases.

^b^The original response options have been collapsed into the presented categories.

^c^28 participants of the nonclinical control group provided contradictory information at a later date. The first statement is reported here.

^d^Based on 279 participants.

^e^Based on 221 participants.

^f^Kruskal–Wallis test.

^g^Based on 713 participants.

^h^Based on 376 participants.

^i^Based on 211 participants.

^j^Based on 63 participants.

^k^Based on 63 participants.

^l^Detailed information on the pairwise comparisons is available in Supporting Information: Tables S1 and S2 of the Supporting Information.

**Table 2:** Clinical characteristics and comparison of the subgroups.

Characteristic	Total sample (*N* = 731)	Nonclinical control group (*n* = 377)	Depression (*n* = 223)	Anxiety disorders (*n* = 65)	Depression and anxiety disorders (*n* = 66)	Univariate between‐subjects tests for subgroups		
	%	%	%	%	%	χ^2^ (df)	*p*	Sign. diff in test comparison^γ^
Number of diagnoses	—	—	—	—	—	*G* = 1117.7 (9)α	<0.001	CG ≠ D CG ≠ A CG ≠ D&A D ≠ D&A A ≠ D&A
None	51.57	100.00	0	0	0	—	—	
One	22.71	0	58.30	55.38	0	—	—	
Two	15.18	0	28.25	27.69	45.45	—	—	
≥Three	10.53	0	13.45	16.92	54.55	—	—	—
Previous psychological treatment								
Previous outpatient treatment	42.03^a^	13.53	66.22^b^	90.63^c^	76.92^d^	273.5 (3)	<0.001	CG < D CG < A CG < D&A D < A A > D&A
Previous inpatient treatment	26.54	2.92	47.53	58.46	59.09	228.16 (3)	<0.001	CG < D CG < A CG < D&A
Current treatment								
Psychological treatment^c^	29.55^f^	0.00	66.07^g^	74.58^h^	71.43^i^	370.44 (3)	<0.001	CG < D CG < A CG < D&A
Pharmacological treatment	28.12^j^	2.39	57.47^k^	47.69	57.58	258.31 (3)	<0.001	CG < D CG < A CG < D&A
	*M* (*SD*)	*M* (*SD*)	*M* (*SD*)	*M* (*SD*)	*M* (*SD*)	—	—	—
Global symptom severity (BSI‐18)	12.66 (12.81)	3.17 (4.14)	21.66 (10.10)	19.43 (9.61)	29.77 (12.70)	497.47 (3)^β^	<0.001	CG < D CG < A CG < D&A D < D&A A < D&A
Sleep quality (PSQI)	6.88 (4.27)^l^	4.31 (2.67)	9.56 (3.71)^m^	8.75 (4.25)^n^	11.52 (3.47)^o^	313.10 (3)^β^	<0.001	CG < D CG < A CG < D&A D < D&AA < D&A
Perceived stress (PSS)	28.16 (8.89)^p^	21.49 (5.70)^q^	35.64 (4.90)^*r*^	32.05 (5.80)	37.26 (5.90)	451.69 (3)^β^	<0.001	CG < D CG < A CG < D&A D > A A < D&A
Depressive symptoms (PHQ‐9)	8.18 (6.96)^s^	2.73 (2.94)^t^	14.33 (4.51)	10.34 (5.25)^c^	16.39 (4.71)	495.87 (3)^β^	<0.001	CG < D CG < A CG < D&A D > A A < D&A
Anxiety symptoms (GAD‐7)	6.26 (5.74)^s^	1.96 (2.10)^t^	10.33 (4.63)	10.31 (5.03)	13.11 (4.29)^d^	469.93 (3)^β^	<0.001	CG < D CG < A CG < D&A D < D&A A < D&A
PTSD symptoms (PCL‐5)	16.25 (17.69)^a^	3.32 (4.78)^t^	27.24 (14.01)^b^	26.52 (16.24)	43.25 (15.12)^d^	479.85 (3)^β^	<0.001	CG < D CG < A CG < D&A D < D&A A < D&A
ER difficulties (DERS)	86.14 (29.79)	64.54 (15.94)^v^	109.16 (21.69)^*r*^	101.17 (25.29)^d^	117.33 (22.95)	426.71 (3)^β^	<0.001	CG < D CG < ACG < D&AA < D&A
Positive affect (PANAS)	2.75 (0.86)^u^	3.32 (0.63)	2.09 (0.57)^w^	2.48 (0.57)	1.96 (0.66)	374.83 (3)^β^	<0.001	CG > D CG > A CG > D&A D < A A > D&A
Negative affect (PANAS)	2.02 (0.89)^a^	1.39 (0.41)	2.68 (0.73)^b^	2.55 (0.76)^c^	2.93 (0.76)^d^	437.83 (3)^β^	<0.001	CG < D CG < A CG < D&A
Illness duration (months)	33.49 (49.8)^x^	—	32.15 (54.7)^y^	35.02 (39.91)^z^	36.57 (40.01)^d^	3.29 (2)^β^	0.193	—

Abbreviations: A, Anxiety disorders; BSI‐18, Brief symptom inventory, PSQI, Pittsburgh sleep quality index; CG, Nonclinical control group; D&A, Depression and anxiety disorders; D, Depression; GAD‐7, Generalized anxiety disorder scale; PANAS, Positive and negative affect schedule; PCL‐5, PTSD Checklist for DSM‐5; PHQ‐9, Patient Health Questionnaire‐9; PSS, Perceived stress scale.

^a^Based on 728 participants.

^b^Based on 222 participants.

^c^Based on 64 participants.

^d^Based on 65 participants.

^e^An error in the questionnaire regarding current psychological treatment has resulted in a high number of missing responses.

^f^Based on 660 participants.

^g^Based on 168 participants.

^h^Based on 59 participants.

^i^Based on 56 participants.

^j^Based on 721 participants.

^k^Based on 221 participants.

^l^Based on 712 participants.

^m^Based on 213 participants.

^n^Based on 60 participants.

^o^Based on 62 participants.

^p^Based on 725 participants.

^q^Based on 374 participants.

^r^Based on 220 participants.

^s^Based on 729 participants.

^t^Based on 376 participants.

^u^Based on 727 participants.

^v^Based on 375 participants.

^w^Based on 219 participants.

^x^Based on 330 participants.

^y^Based on 211 participants.

^z^Based on 54 participants.

^α^G‐Test.

^β^Kruskal–Wallis test.

^γ^Detailed information on the pairwise comparisons is available in Supporting Information: Tables S1 and S2.

As data from the clinical groups were collected as part of a larger project, the sample size was determined independently of the current study. However, we conducted a power analysis (using *G* ^∗^Power) to assess whether the sample size was sufficient to detect the expected effects. The power analysis was based on a 2 × 2 ANOVA with an alpha = 0.008 (Bonferroni corrected for the number of subscales, namely six), assuming a power of 0.80 and a medium effect size of *f* = 0.25. Results indicated that a total sample of *N* = 199 participants, with 50 participants per group (depression only, anxiety only, depression and anxiety, and nonclinical controls), would be required to detect the expected effect size.

### 2.2. Measures

#### 2.2.1. Structured Clinical Interview for DSM‐5‐Clinician Version (SCID‐5‐CV)

The SCID‐5‐CV ([34]; German version: [35]) was administered to determine inclusion and exclusion diagnoses. The SCID‐5‐CV is a semistructured instrument designed to assess disorders according to the Diagnostic and Statistical Manual of Mental Disorders, 5th edition (DSM‐5; American Psychiatric [36]). The SCID‐5‐CV was supplemented with additional modules from the SCID‐5 Research Version, covering somatic symptom and related disorders, specific phobia, sleep disorders, feeding and eating disorders, body dysmorphic disorder, gambling disorder, and intermittent explosive disorder. The SCID‐5‐CV demonstrates adequate psychometric properties, with positive agreement rates between the interview and clinical diagnoses ranging from 73% to 97%, diagnostic sensitivity and specificity > 0.70, and kappa > 0.70 for most diagnoses [37].

#### 2.2.2. Demographic Questionnaire

The demographic questionnaire collected information on general characteristics such as age, gender, marital status, and socioeconomic status. Additionally, it included questions about medication use and psychological treatment. Self‐reported physical activity in minutes per week was assessed using the Physical Activity, Exercise, and Sport Questionnaire (BSA questionnaire; [38]).

#### 2.2.3. DERS

The DERS ([14]; German version: [39]) was used to assess self‐reported ER difficulties. The 36‐item scale yields a total score as well as six subscale scores, including *Nonacceptance of Emotional Responses* (6 items; e.g., “When I’m upset, I become embarrassed for feeling that way.”), *Difficulties Engaging in Goal-Directed Behavior* (5 items; e.g., “When I’m upset, I have difficulty focusing on other things.”), *Impulse Control Difficulties* (6 items; e.g., “I experience my emotions as overwhelming and out of control.”), *Lack of Emotional Awareness* (6 items; e.g., “I am attentive to my feelings.”, reverse‐scored), *Limited Access to ER Strategies* (8 items; e.g., “When I’m upset, I believe that I will end up feeling very depressed.”), and *Lack of Emotional Clarity* (5 items; e.g., “I have no idea how I am feeling.”). Participants rated each item on a 5‐point Likert scale ranging from 1 (*almost never*, *0%–10%*) to 5 (*almost always*, *91%–100%*), with higher scores indicating greater ER difficulties. The DERS demonstrates good psychometric properties for all subscales with internal consistencies *αs* > 0.80 and test–retest reliability *r* = 0.57 to 0.89 [14]. In this study, Cronbach’s alpha was 0.93 for the clinical sample, 0.91 for the nonclinical sample, and 0.96 for the total sample.

#### 2.2.4. PTQ

The PTQ is a 15‐item self‐report questionnaire that assesses transdiagnostic RNT [83]. Items were rated on a 5‐point Likert scale ranging from 0 (*never*) to 4 (*almost always*), with higher scores indicating a greater frequency of RNT. The PTQ shows good internal consistency with *αs* > 0.77 and satisfactory test–retest reliability *r* = 0.69 [83]. In this study, Cronbach’s alpha was 0.94 for the clinical sample, 0.95 for the nonclinical sample, and 0.98 for the total sample.

#### 2.2.5. Symptom Questionnaires

To assess symptom severity, a comprehensive set of instruments was employed. The *Brief Symptom Inventory* (BSI‐18; [40]; German version: [41]) was used to evaluate global symptom severity, along with the severity of somatic, depressive, and anxiety symptoms. Depressive symptoms were specifically assessed using the *Patient Health Questionnaire-9* (PHQ‐9; [42]; German version: [43]), while the seven‐item *Generalized Anxiety Disorder Scale* (GAD‐7; [44]; German version: [45]) was used to measure the severity of generalized anxiety symptoms. Additionally, symptoms of PTSD were measured using the *PTSD Checklist for DSM*‐5 (PCL‐5; [46]; German version: [47]). For the global assessment of emotional sensitivity, the *Positive and Negative Affect Schedule* (PANAS; [48]; German version: [49]) was administered. The *Pittsburgh Sleep Quality Index* (PSQI; [50]) was used to assess sleep quality. Stress was assessed with the *Perceived Stress Scale* (PSS; [51]). All questionnaires have demonstrated good internal consistency and adequate test–retest reliability. In this study, Cronbach’s alpha values ranged from 0.80 to 0.92 for the clinical sample, from 0.73 to 0.87 for the nonclinical sample, and from 0.92 to 0.96 for the total sample.

### 2.3. Procedure

Potential participants for the clinical group initially underwent a telephone screening to assess whether they met the inclusion criteria for the ImPuls project. Those who successfully passed the screening attended a scheduled appointment during which written informed consent was obtained. The SCID‐5‐CV was then administered, either face‐to‐face or via videoconferencing, to confirm eligibility. Interviews were conducted by master’s‐level psychologists undergoing advanced training in cognitive behavioral therapy. After eligibility was confirmed, six individuals from the same site were grouped together; they received a link to an online assessment with a more extensive battery of questionnaires as part of the overarching study. This set of questionnaires, including those described above, was to be completed within a 14‐day period. Compensation for participation in the clinical group was contingent on assignment to either treatment as usual (TAU) or the active treatment group. However, at the time of collecting baseline data for this study, participants had not undergone randomization, and compensation had not yet been determined or disbursed. After completing all assessments in the parent study, participants in the control group received compensation amounting to €450^2^. Individuals expressing interest in joining the nonclinical control group initiated participation by completing a brief online screening, in which inclusion and exclusion criteria were assessed. This screening also gathered relevant information for matching (age, gender, and education). Those who met the inclusion criteria and were successfully matched with a clinical participant were then interviewed using the SCID‐5‐CV, conducted by graduate psychology students via videoconferencing. These assessors underwent thorough training and were supervised by the authors. Participants who were not diagnosed with any mental disorder that would lead to exclusion were provided with a link to the same online survey as the clinical sample. Participants in the nonclinical control group received compensation of 8€ per hour or course credit upon completion of the assessment.

This study was approved by the ethics committee for medical research at the University of Tübingen (ID: 888/2020B01), and all procedures were conducted in accordance with the committee’s ethical standards and with the 1964 Declaration of Helsinki and its later amendments. The study was preregistered with the Open Science Framework https://osf.io/e35hg.

### 2.4. Data Analyses

To assess differences in the different ER difficulties, we ran a 2 × 2 MANOVA (as preregistered) with depression (presence vs. absence) and anxiety (presence vs. absence) as between‐subjects factors on the six subscales of the DERS as the dependent variables. We relied on Pillai’s Trace, as the normality and homoscedasticity assumptions appeared to be violated [52]. To address potential biases, we also conducted a complementary semiparametric repeated‐measures MANOVA as a robustness check. The ANOVA‐type statistic (ATS) was employed for the semiparametric MANOVA. If the MANOVAs indicated significant effects, we performed subsequent post‐hoc tests. For the parametric approach, Bonferroni–Holm (BH)‐corrected^3^ ANOVAs were conducted, followed by BH‐corrected pairwise comparisons to examine specific group comparisons. Additionally, as a nonparametric alternative, BH‐corrected Scheirer–Ray–Hare tests were performed, followed by BH‐corrected Dunn’s tests.

Similarly, for the PTQ scores, a 2 (depression: presence vs. absence) x2 (anxiety: presence vs. absence) ANOVA was initially planned. However, due to the violation of the normality assumption, we supplemented the planned parametric ANOVA with a nonparametric Scheirer–Ray–Hare test to confirm the findings using a combined parametric and nonparametric approach. When significant differences emerged, post‐hoc BH‐corrected *t*‐tests and BH‐corrected Dunn’s tests were performed for pairwise comparisons.

In addition, we conducted a sensitivity analysis to examine whether group differences in RNT remained when accounting for shared variance with overall ER difficulties. For this purpose, we estimated an ANCOVA model in which PTQ scores were predicted by depression (presence vs. absence), anxiety (presence vs. absence), and their interaction, with the DERS total score included as a covariate. This analysis was conducted to assess the robustness of the observed group differences in RNT while acknowledging the substantial conceptual and empirical overlap between ER difficulties and RNT. Given the cross‐sectional nature of the data, this analysis was interpreted descriptively, and no causal conclusions were drawn.

To explore the effect of the number of comorbid diagnoses on DERS subscales, a MANOVA was conducted with four groups (number of diagnoses: no diagnosis, one diagnosis, two diagnoses, and three or more diagnoses) as the between‐subjects factor and the six DERS subscales as dependent variables. This grouping approach for the number of diagnoses was selected based on the precedent set by McEvoy et al. [25]. Additionally, this categorization ensured sufficient statistical power by maintaining adequate sample sizes within each group for meaningful comparisons. Given the assumption violations of normality and homogeneity of variance, a corresponding semiparametric repeated‐measures MANOVA was also performed. Post‐hoc tests included BH‐corrected ANOVAs with post‐hoc *t*‐tests and nonparametric BH‐corrected Welch’s ANOVA with Games–Howell post‐hoc tests. For the PTQ scores, a one‐way ANOVA was planned. However, since the normality assumption was violated, the Kruskal–Wallis test was used instead with BH‐corrected Dunn’s test for post‐hoc comparison. Comorbid disorders, in this context, referred to all disorders currently present and assessed through the SCID interview.

Because no suitable implementation in R was available for handling multiple imputed datasets in semiparametric analyses, and because the number of missing values was very small, we deviated from our preregistration and opted to use a single dataset, excluding missing values. This decision aimed to ensure the robustness of our findings while acknowledging the potential limitations it may introduce. All statistical analyses were conducted using R (Version 4.4.1; R Core [53]).

### 2.5. Use of AI‐Assisted Tools

During the preparation of this manuscript, the authors used ChatGPT (OpenAI, large language model) to assist with language editing, grammar refinement, and improving clarity of expression. The generated text was critically reviewed and revised by the authors, who take full responsibility for the final content of the manuscript.

## 3. Results

### 3.1. Participant Characteristics

The sociodemographic and clinical characteristics of the sample are presented in Tables 1 and 2. Detailed results of the post‐hoc comparisons and standardized residuals are provided in the Supporting Information (Supporting Information: Tables S1 and S2). Significant differences were observed in the gender distribution across groups. Specifically, the control group and the depression group had a significantly lower proportion of individuals who did not identify as female or male compared to the anxiety group. Additionally, the mixed group had significantly fewer men than the control and depression groups, and a higher proportion of women compared to the depression group. Employment status also differed significantly between the control group and the three clinical groups. A higher proportion of individuals in the clinical groups were unemployed or employed less often compared to the control group. Moreover, the mixed group had a significantly higher proportion of individuals categorized as “other” in terms of employment status than the control group. In terms of physical activity, the depression group reported significantly less activity than both the anxiety group and the control group. As expected, the control group differed significantly from all clinical groups across all clinical characteristics. The mixed group displayed a significantly higher number of diagnoses compared to both the anxiety and depression groups. This group also had significantly more previous outpatient treatments, greater global symptom severity (BSI‐18), poorer sleep quality (PSQI), higher PSS, more severe anxiety symptoms (GAD‐7), more severe PTSD symptoms (PCL‐5), and lower positive affect (PANAS) than the anxiety group. When comparing the mixed group to the depression group, the mixed group exhibited significantly PSQI, more severe anxiety symptoms (GAD‐7), and more pronounced PTSD symptoms (PCL‐5). The anxiety group, on the other hand, had significantly more previous outpatient treatments than the depression group. The depression group reported significantly higher PSS, more severe depressive symptoms (PHQ‐9), and greater negative affect (PANAS) compared to the anxiety group. Lastly, illness duration did not differ significantly between the clinical groups. Given that age also did not differ between groups, this suggests that age of onset is unlikely to vary systematically across diagnostic groups.

### 3.2. ER Across Diagnostic Groups

The 2 × 2 MANOVA on the DERS subscales revealed significant main effects of depression, Pillai’s Trace = 0.29, *F*(6,717) = 48.90, *p* < 0.001; and anxiety, Pillai’s Trace = 0.17, *F*(6,717) = 25.06, *p* < 0.001, as well as a significant interaction, Pillai’s Trace = 0.09, *F*(6,717) = 12.40, *p* < 0.001. The semiparametric repeated‐measures MANOVA confirmed these findings, MATS = 124.82, *df* = 6, *p* < 0.001, indicating the robustness of the results against assumption violations. Subsequent BH‐corrected ANOVAs and nonparametric Scheirer–Ray–Hare tests indicated significant group differences (Table 3 and Supporting Information: Table S3).

**Table 3:** Means, standard deviations, and results of MANOVA, ANOVAs, and post‐hoc *t*‐tests for emotion regulation difficulties subscales and repetitive negative thinking—comparison of the subgroups.

Variable	Group				Test of between‐subjects effects/ANOVA	Significant differences in post‐hoc comparisons
	Nonclinical control group *M* (SD)	Depression *M* (SD)	Anxiety disorders *M* (SD)	Depression and anxiety disorders *M* (SD)		
DERS: nonacceptance	10.15 (4.12)^a^	17.95 (5.66)^c^	16.62 (6.98)^e^	19.85 (5.62)^f^	*F*_(1, 722)_ = 21.57 ^∗∗∗^	D&A > D, A > CG
DERS: goals	10.25 (3.78)^a^	17.45 (4.04)^c^	16.09 (4.83)^e^	19.23 (4.31)^f^	*F*_(1,722)_ = 27.31 ^∗∗∗^	D&A > D > A > CG
DERS: impulse	9.12 (3.17)^a^	14.32 (4.92)^c^	14.49 (5.86)^e^	17.09 (6.11)^f^	*F*_(1,722)_ = 9.50 ^∗∗^	D&A > D, A > CG
DERS: awareness	14.49 (4.56)^a^	19.88 (4.60)^c^	18.38 (4.81)^e^	19.23 (5.36)^f^	*F*_(1,722)_ = 25.10 ^∗∗∗^	D&A, D, A > CG
DERS: strategies	12.39 (4.29)^a^	25.57 (6.96)^c^	22.89 (7.45)^e^	27.80 (6.95)^f^	*F*_(1,722)_ = 54.14 ^∗∗∗^	D&A > D > A > CG
DERS: clarity	8.15 (2.76)^a^	13.98 (4.57)^c^	12.69 (4.76)^e^	14.14 (4.78)^f^	*F*_(1,722)_ = 35.48 ^∗∗∗^	D&A, D, A > CG
PTQ	14.81 (11.41)^b^	39.39 (10.53)^d^	37.26 (11.50)^e^	43.49 (9.57)^e^	*F*_(1,718)_ = 73.17 ^∗∗∗^	D&A > D, A > CG

Abbreviations: A, Anxiety disorders; CG, Nonclinical control group; D, Depression; D&A, Depression and anxiety disorders; DERS, Difficulties in emotion regulation scale; PTQ, Perseverative Thinking Questionnaire.

^a^Based on 375 participants.

^b^Based on 373 participants.

^c^Based on 220 participants.

^d^Based on 219 participants.

^e^Based on 65 participants.

^f^Based on 66 participants.

^∗^ *p* < 0.05.  ^∗∗^ *p* < 0.01.  ^∗∗∗^ *p* < 0.001.

Confirming Hypotheses 1a and 2a, the post‐hoc BH‐corrected pairwise *t*‐tests revealed that, on all subscales of the DERS, the nonclinical control group scored significantly lower than the anxiety group (Cohen’s *d* range: 1.39–2.15), the depression group (Cohen’s *d* range: 1.33–2.43), and the mixed group (Cohen’s *d* range: 2.12–3.23). The nonparametric Dunn’s tests confirmed these results (Supporting Information: Tables S5 and S7).

Regarding Hypothesis 3, the post‐hoc BH‐corrected pairwise *t*‐tests yielded different results between the mixed group and the depression group and anxiety group, depending on the DERS subscale.

On the *nonacceptance of emotional responses* subscale, the mixed group showed significantly greater difficulties than the anxiety group (Cohen’s *d* = 0.51) and the depression group (Cohen’s *d* = 0.34). However, using the nonparametric approach, only the difference compared to the anxiety group remained significant. The exploratory analyses revealed no difference between the anxiety and the depression groups.

On the *difficulties engaging in goal-directed behavior* subscale, the mixed group exhibited significantly greater difficulties than the anxiety group (Cohen’s *d* = 0.69) and the depression group (Cohen’s *d* = 0.43). However, using the nonparametric approach, only the difference compared to the anxiety group, but not to the depression group remained significant. Furthermore, the exploratory analyses revealed significantly greater difficulties in the depression group compared to the anxiety group (Cohen’s *d* = 0.32). However, when the more conservative Dunn’s test was applied, this difference was no longer significant.

On the *impulse control difficulties* subscale, the mixed group showed significantly greater difficulties than the anxiety group (Cohen’s *d* = 0.43) and the depression group (Cohen’s *d* = 0.53). However, neither comparison remained significant using the nonparametric approach. The exploratory analyses showed no significant differences between the anxiety and the depression groups.

On the *lack of emotional awareness* subscale, no significant differences between any of the three clinical groups were found using either the parametric or the nonparametric approach.

On the *limited access to ER strategies* subscale, the mixed group showed significantly greater difficulties than both the anxiety group (Cohen’s *d* = 0.68) and the depression group (Cohen’s *d* = 0.32). However, using the nonparametric approach, only the comparison between the mixed group and the anxiety group remained significant. Furthermore, the exploratory analyses showed significantly greater difficulties in the depression group compared to the anxiety group (Cohen’s *d* = 0.38). However, when the more conservative Dunn’s test was applied, this difference was no longer significant.

On the *lack of emotional clarity* subscale, no significant differences between any of the three clinical groups were found using either the parametric or the nonparametric approach.

### 3.3. RNT Across Diagnostic Groups

The 2 × 2 ANOVA on the PTQ scores revealed significant main effects of depression, *F*(1, 718) = 688.69, *p* < 0.001, and anxiety, *F*(1, 718) = 230.54, *p* < 0.001, as well as a significant interaction. The Scheirer–Ray–Hare test confirmed these findings, MATS = 2882.60, *df* = 18, *p* < 0.001 (Table 3 and Supporting Information: Table S3).

Post‐hoc BH‐corrected *t*‐tests showed that the anxiety group (Cohen’s *d* = 1.97), the depression group (Cohen’s *d* = 2.22), and the mixed group (Cohen’s *d* = 2.57) all had significantly higher PTQ scores compared to the nonclinical control group. Dunn’s tests confirmed these results (Supporting Information: Table S5 and S7).

**Figure 1:**
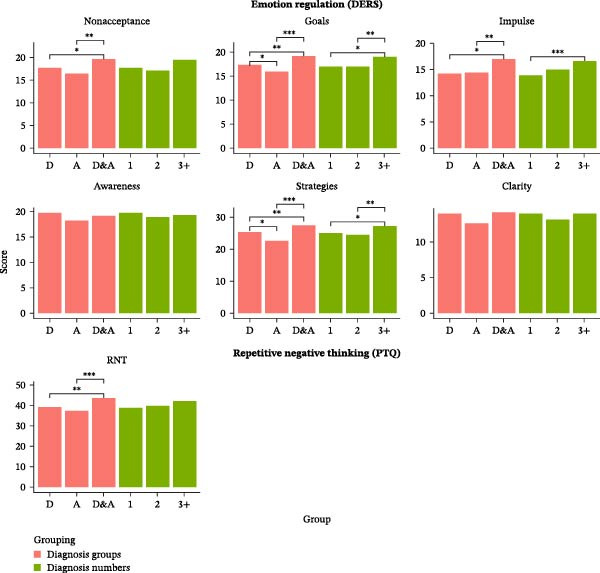
Comparison of emotion regulation subscales and repetitive negative thinking across diagnostic groups and number of diagnoses. Abbreviations: 1, one diagnosis; 2, two diagnoses; 3+, three or more diagnoses; *A*, Anxiety; *D*, Depression; D&A, Depression and anxiety; PTQ, Perseverative Thinking Questionnaire.  ^∗^ *p* < 0.05.  ^∗∗^ *p* < 0.01.  ^∗∗∗^ *p* < 0.001.

In a comparison between the diagnostic groups, the mixed group showed significantly higher values than the anxiety group (Cohen’s *d* = 0.59) and the depression group (Cohen’s *d* = 0.40; see Figure 1). However, these differences were no longer significant when Dunn’s test was applied.

As a sensitivity analysis, we conducted an ANCOVA in which overall ER difficulties (DERS total score) were included as a covariate in the model predicting PTQ scores. The main effects of depression, *F* (1,717) = 84.41, *p*  < 0.001, and anxiety, *F* (1,717) = 58.96, *p*  < 0.001, as well as their interaction, *F* (1,717) = 25.88, *p*  < 0.001, remained significant. ER difficulties were strongly associated with RNT, *F* (1,717) = 363.23, *p*  < 0.001 (see Supporting Information: Table S9). Post‐hoc comparisons based on estimated marginal means indicated that all clinical groups continued to show significantly higher RNT levels than nonclinical controls after adjusting for ER difficulties (all *p*  < 0.001). In contrast, differences between the anxiety, depression, and comorbid groups were no longer significant when ER difficulties were included as a covariate (see Supporting Information: Table S10).

### 3.4. Relationship Between Number of Comorbidities and ER

The MANOVA on the number of comorbid diagnoses and DERS subscales revealed a significant main effect, Pillai’s Trace = 0.63, *F*(18, 2157) = 31.81, *p* < 0.001. The semiparametric repeated‐measures MANOVA confirmed these results (Table 4 and Supporting Information: Table S4).

**Table 4:** Means, standard deviations, and results of MANOVA, ANOVAs, and post‐hoc *t*‐tests for emotion regulation difficulties subscales and repetitive negative thinking—comparison of the number of diagnoses.

Variable	Group					
	No diagnosis *M* (*SD*)	1 diagnosis *M* (*SD*)	2 diagnoses *M* (*SD*)	3+ diagnoses *M* (*SD*)	Test of between‐subjects effects/ANOVA	Significant differences in post‐hoc comparisons
DERS: nonacceptance	10.15 (4.12)^a^	17.93 (5.66)^c^	17.27 (6.28)^e^	19.49 (6.06)^g^	*F*_(3, 722)_ = 149.36 ^∗∗∗^	3+, 2, 1, > 0
DERS: goals	10.25 (3.78)^a^	17.13 (4.07)^c^	17.06 (4.71)^e^	19.11 (4.05)^g^	*F*_(3, 722)_ = 203.06 ^∗∗∗^	3 + > 2,1 > 0
DERS: impulse	9.12 (3.17)^a^	13.96 (4.93)^c^	15.03 (5.84)^e^	16.65 (5.48)^g^	*F*_(3, 722)_ = 112.17 ^∗∗∗^	3 + > 1; 3+, 2,1 > 0
DERS: awareness	14.49 (4.55)^a^	19.87 (4.73)^c^	18.95 (4.79)^e^	19.42 (5.00)^g^	*F*_(3, 722)_ = 69.54 ^∗∗∗^	3+, 2, 1 > 0
DERS: strategies	12.39 4.29)^a^	25.18 (6.91)^c^	24.60 (7.74)^e^	27.47 (6.68)^g^	*F*_(3, 722)_ = 308.98 ^∗∗∗^	3+ > 2, 1 > 0
DERS: clarity	8.15 (2.76)^a^	13.99 (4.63)^c^	13.23 (4.78)^e^	14.08 (4.54)^g^	*F*_(3, 722)_ = 133.52 ^∗∗∗^	3+, 2, 1 > 0
PTQ	14.81 (11.41)^b^	38.87 (9.90)^d^	39.55 (12.03)^f^	41.96 (10.16)^g^	*F*_(3, 718)_ = 307.44 ^∗∗∗^	3+, 2, 1 > 0

Abbreviations: 0, no diagnosis; 1, one diagnosis; 2, two diagnoses; 3+, three or more diagnoses; DERS, Difficulties in emotion regulation scale; PTQ, Perseverative Thinking Questionnaire.

^a^Based on 375 participants.

^b^Based on 373 participants.

^c^Based on 165 participants.

^d^Based on 164 participants.

^e^Based on 110 participants.

^f^Based on 109 participants.

^g^Based on 76 participants.

^∗^ *p* < 0.05.  ^∗∗^ *p* < 0.01.  ^∗∗∗^ *p* < 0.001.

BH‐corrected follow‐up ANOVAs and subsequent post‐hoc tests indicated significant differences between the group with no diagnosis and the other three groups with one, two, and three or more diagnoses on all subscales of the DERS, with the group with no diagnoses showing fewer emotional regulation difficulties (Cohen’s *d* range: 0.79–3.16; Supporting Information: Table S6). For the subscales of *nonacceptance of emotional responses*, *lack of emotional awareness*, and *lack of emotional clarity*, no further significant differences were found between any of the diagnostic groups. For the subscale *difficulties engaging in goal-directed behavior*, significant differences were found between the group with one diagnosis and the group with three or more diagnoses (Cohen’s *d* = 0.48), as well as between the group with two diagnoses and the group with three or more diagnoses (Cohen’s *d* = 0.46). For the *impulse control difficulties* subscale, the comparison between the group with one diagnosis and the group with three or more diagnoses was also significant (Cohen’s *d* = 0.53). For the *limited access to ER strategies* subscale, the group with one diagnosis and the group with two diagnoses showed significantly fewer difficulties than the group with three or more diagnoses (Cohen’s *d* = 0.34 and 0.39). However, the difference between the group with one diagnosis and the group with three or more diagnoses was no longer significant when the Games–Howell test was used (Supporting Information: Table S8).

### 3.5. Relationship Between Number of Comorbidities and RNT

A one‐way ANOVA showed a significant effect of the number of comorbid diagnoses on PTQ scores. The Kruskal–Wallis test, as a nonparametric alternative, also indicated significant differences (Table 4 and Supporting Information: Table S4).

Post‐hoc BH‐corrected *t*‐tests revealed that only individuals with no diagnosis had significantly lower PTQ scores than individuals with one (Cohen’s *d* = 2.19), two (Cohen’s *d* = 2.24), or three or more diagnoses (Cohen’s *d* = 2.42). No significant differences were found between the groups with at least one diagnosis (see Figure 1). The Kruskal–Wallis and post‐hoc Dunn’s tests confirmed these results (Supporting Information: Tables S6 and S8).

The detailed results of the additional semiparametric and nonparametric tests can be found in the Supporting Information (Supporting Information: Tables S3, S4, S7, and S8). Figure 1 provides an overview of the comparisons between the clinical groups. In all analyses, gender was controlled for by including it as a covariate in the statistical models. The inclusion of gender did not alter any of the findings: significant differences between groups remained significant, and nonsignificant results remained nonsignificant. This indicates that the observed effects are robust and not influenced by gender differences.

## 4. Discussion

In this study, we investigated differences in ER difficulties and RNT between nonclinical individuals and those diagnosed with depression, anxiety disorders, or both. Our goal was to determine whether ER difficulties are specific to certain disorders or transdiagnostic and whether greater difficulties are associated with a higher number of comorbidities.

As hypothesized, individuals with either an anxiety disorder or a depression reported significantly greater difficulties in all dimensions of ER compared to nonclinical controls. This finding aligns with previous research indicating that ER difficulties are common across various mental health disorders (e.g., [15, 54, 55]). These results underscore the transdiagnostic nature of ER difficulties, suggesting that these difficulties are not merely artifacts of specific diagnostic categories but rather a pervasive feature across multiple forms of psychopathology. The greater difficulties observed in the anxiety group and the depression group compared to nonclinical controls support the idea that emotional dysregulation may be a core component of these disorders. Notably, unlike Shukla and Pandey [4], who did not observe differences between the control group and the anxiety group or the mixed group on all DERS scales, our findings highlighted more pronounced difficulties. This discrepancy may be due to the inclusion of a clinical sample with more severe symptomatology in our study, reinforcing the importance of considering clinical severity when examining ER difficulties.

Our study also highlighted the significance of RNT as a transdiagnostic process. As hypothesized, both the anxiety group and the depression group, as well as the mixed group, exhibited higher levels of RNT compared to nonclinical controls, which is consistent with existing literature (e.g., [23, 25]). The consistent elevation of RNT across all clinical groups suggests that this cognitive process may serve as a common pathway linking different mental health conditions. This pattern is particularly relevant for clinical interventions, as targeting RNT could potentially mitigate symptoms across a range of disorders. Sensitivity analyses provided additional nuance to these findings. Whereas the primary analyses indicated higher levels of RNT in the comorbid group compared to the single‐diagnosis groups, these between‐clinical‐group differences were attenuated when overall ER difficulties were included as a covariate. Importantly, all clinical groups continued to show elevated RNT relative to nonclinical controls. This pattern suggests that the additional elevation of RNT observed in individuals with comorbid conditions overlaps substantially with overall ER difficulties, rather than reflecting an entirely independent effect of comorbidity. A complementary pattern has been observed in the same outpatient cohort, where both global ER difficulties and RNT explained unique variance in global symptom severity within a hierarchical regression model [56]. When examined within the same statistical framework, both constructs retained statistically independent associations with symptom severity. Together, these findings suggest that ER difficulties and RNT share substantial conceptual overlap while also representing distinguishable transdiagnostic processes.

Regarding our hypotheses that the presence of comorbidities is associated with greater ER difficulties and RNT, it is important to note that we conducted both parametric and nonparametric analyses to examine the differences between the various groups. In some cases, the parametric analyses showed significant effects, whereas the nonparametric tests did not confirm these findings. Since the assumptions for parametric tests were not met, their results should be interpreted with caution. Nonparametric analyses, though more robust against assumption violations, have lower statistical power, which may lead to nonsignificant results due to decreased sensitivity. Consequently, the most robust findings are those supported by both parametric and nonparametric tests. Results that emerged only from the parametric tests, as well as findings from our exploratory analyses, should be interpreted cautiously and replicated in future studies.

When comparing the mixed, anxiety, and depression groups (Hypothesis 3 and exploratory analyses), as well as differences based on the number of comorbidities (Hypothesis 4), no significant differences were found in lack of emotional awareness and clarity. Deficits in emotional awareness (attention to emotional responses) and emotional clarity (understanding and identifying emotions) were equally present in individuals with depression, anxiety disorders, and comorbid depression and anxiety. Moreover, these difficulties were consistent regardless of the number of comorbid diagnoses. These results align with the findings of Shukla and Pandey [4], who likewise did not observe differences between the anxiety group and the mixed group on these subscales.

Regarding the lack of differences in emotional awareness and clarity across diagnostic groups and comorbidity levels, several explanations may account for this pattern. The consistency of deficits in emotional awareness and emotional clarity across clinical groups and levels of comorbidity suggests that these difficulties are fundamental issues across anxiety disorders and depression, not just a consequence of more complex diagnostic profiles. Further studies are necessary to elucidate whether these deficits function as risk factors, maintaining factors, or consequences of altered emotionality. Some evidence pointing to a risk or maintaining factor exists, particularly in disorders characterized by significant emotional changes. For instance, low emotional awareness has been identified as a transdiagnostic mechanism in females, contributing to the development of psychopathology during the transition to adolescence, especially following childhood trauma [57]. Blöte and Westenberg [58] showed that depressive symptoms and low emotional clarity tend to predict each other’s increase during adolescence over time, suggesting a vicious cycle between a lack of emotional clarity and depressive symptoms. Both deficits could also be linked to experiential avoidance, which is associated with increased risk and maintenance of depression and anxiety [59, 60]. All three constructs share the characteristic of limiting an individual’s engagement with their emotional experiences [61]. Low emotional awareness and clarity may lead individuals to avoid emotions, hindering effective regulation and promoting maladaptive coping strategies that, in turn, exacerbate emotional dysregulation. This avoidance further reinforces maladaptive emotional responses and perpetuates psychopathology. Alternatively, difficulties in emotional awareness and clarity might emerge as a result of altered emotionality that characterizes anxiety and depression. The persistent intensity or duration of emotional experiences in these conditions might disrupt how emotions are perceived and categorized, affecting emotional awareness and clarity. For instance, a general emotional dullness in depression and an external focus that shifts attention away from internal states in anxiety might exacerbate these difficulties.

Regarding the remaining four subscales of the DERS and the effects of RNT (PTQ), the results were less consistent. Different patterns emerged

First, the mixed group exhibited greater difficulties on the *nonacceptance of emotional responses* subscale as well as higher RNT than both the anxiety group and depression group. This could indicate that comorbidity, particularly involving both depression and anxiety, amplifies difficulties in accepting emotional responses and contributes to increased RNT. However, comparisons based on the number of diagnoses did not reveal a straightforward increase in difficulties. One potential explanation is that RNT and difficulties with nonacceptance of emotional responses are more closely linked to depression than to anxiety‐related disorders. Indeed, McEvoy et al. [25] found that increased RNT with a higher number of diagnoses was present only in individuals with a primary diagnosis of depression, not in those with a primary diagnosis of an anxiety disorder. As discussed above, additional sensitivity analyses suggest that this pattern overlaps substantially with overall ER difficulties. Although similar research on nonacceptance of emotional responses is lacking, it is plausible that the cognitive and emotional patterns associated with depression, such as heightened self‐criticism and rumination, might be linked to difficulties in accepting emotions. For instance, individuals who struggle to accept their emotional responses might perceive these emotions as overwhelming or unjustified, which could heighten feelings of inadequacy or hopelessness, both of which are common in depression. Moreover, individuals with depression may be more inclined to perceive their emotional responses as a sign of personal failure or weakness, which, in turn, could reinforce nonacceptance. In contrast, anxiety disorders may be more closely associated with avoidance strategies to manage overwhelming emotions, focusing on avoiding situations or feelings that trigger their anxiety. While these avoidance strategies might help reduce immediate emotional distress, they may not directly lead to nonacceptance of emotions in the same way as depressive rumination and self‐criticism. Another explanation may be related to sample characteristics in this study. For instance, we found significantly more PTSD symptoms in the mixed group compared to the anxiety group or the depression group. Previous research has demonstrated that individuals with PTSD exhibit significant difficulties in ER, particularly in terms of nonacceptance of emotional responses and RNT [62–64].

Second, the mixed group also showed higher scores on the *impulse control difficulties* subscale than either the anxiety or depression group. Moreover, impulse control difficulties may contribute to psychopathology, as individuals with at least three diagnoses exhibited greater deficits than those with only one diagnosis. This finding could be interpreted through the lens of the cumulative burden of mental health disorders [65]. Individuals with multiple diagnoses might experience an overload of stressors, exacerbating difficulties in impulse control. The combination of various psychopathological symptoms could lead to a heightened state of emotional dysregulation, making it increasingly challenging for individuals to control impulsive behaviors. For example, in individuals with both anxiety and depression, the co‐occurrence of these disorders may result in a constant state of heightened arousal (due to anxiety) coupled with a pervasive sense of hopelessness (due to depression; [65, 66]). This emotional turmoil might overwhelm the individual’s capacity to regulate impulses effectively. However, the lack of a significant difference between the groups with one and two diagnoses also allows the alternative explanation that the increase in difficulties may be related to the presence of specific diagnoses. The interaction between specific disorders within the mixed group could be contributing to the observed difficulties in impulse control. For instance, the combination of PTSD and depression may uniquely impair impulse control due to the synergistic effects of hyperarousal from PTSD and cognitive impairments from depression. As previous research has shown, PTSD is often linked to impulsive behaviors [67, 68]. When combined with the cognitive rigidity and negative biases commonly associated with depression, the capacity to regulate impulses may be further compromised. Lastly, it is important to consider the role of coping mechanisms in individuals with multiple diagnoses. Maladaptive coping strategies, such as avoidance, are more likely to develop in individuals struggling with multiple mental health challenges. These strategies can further impair impulse control by reducing individuals’ ability to engage in more adaptive forms of ER. Thus, the higher impulse control difficulties observed in the mixed group might also reflect the cumulative impact of maladaptive coping strategies that may develop in response to coping with multiple disorders.

Third, for the *difficulties engaging in goal-directed behavior* and *limited access to ER strategies* subscales, the mixed group displayed greater difficulties than the depression group, which in turn showed more difficulties than the anxiety group. This could indicate that a diagnosis of depression may be associated with or even lead to increased difficulties in these domains. Cognitive models of depression (e.g., [69]) emphasize the role of negative thought patterns and dysfunctional cognitions, which can impair goal‐directed behavior. For example, anhedonia, a core symptom of depression, directly affects motivation and the ability to engage in purposeful activities. Additionally, concentration problems and rumination, both common in depression, can further hinder goal‐directed behavior [70]. Similarly, the *limited access to ER strategies* subscale is likely influenced by the persistent nature of depressive symptoms. Depression is characterized by prolonged periods of low mood, during which individuals often feel unable to change their emotional state (American Psychiatric [36]). This contrasts with conditions like anxiety disorders, where intense emotional states such as panic may be transient. The chronic nature of depression may lead to a sense of helplessness, where individuals perceive fewer options for regulating their emotions effectively, contributing to the higher scores on this subscale. At the same time, these subscales also show significantly higher levels of difficulties as the number of comorbidities increases. This may be due to the cumulative burden of multiple disorders, such as depression, anxiety, and PTSD, which can overwhelm cognitive resources. For example, motivational difficulties and concentration problems in depression may be compounded by the worry and hypervigilance of anxiety, making it harder to engage in goal‐directed behavior. Similarly, the combination of chronic depressive symptoms and the emotional reactivity of anxiety or PTSD can limit access to effective ER strategies, leading to higher difficulties. However, methodological factors related to sample characteristics could also explain the observed pattern. It is possible that the depression group includes a higher proportion of individuals with more severe or chronic symptoms, which could explain the greater symptom severity on some of the assessed clinical scales compared to the anxiety group. Consequently, the results for these ER difficulties subscales may reflect a correlation with overall pathology. To address this, future studies should employ more objective measures of ER to minimize potential biases from self‐report data and to ensure that observed differences reflect genuine effects rather than artifacts of sample composition.

Beyond these specific diagnostic explanations, other underlying mechanisms may also contribute to the observed differences. For instance, neurobiological factors, such as altered brain connectivity, may influence ER (e.g., [71, 72]). Psychological factors, such as attentional biases toward negative stimuli and reduced cognitive flexibility, could further impair ER [73, 74]. Environmental factors, such as chronic stress or trauma exposure, might also play a moderating role, leading to greater challenges in ER irrespective of specific diagnoses [75, 76]. Finally, personality traits such as high neuroticism or low emotional resilience may predispose individuals to greater emotional dysregulation, particularly in the presence of comorbid conditions [77, 78]. These factors could help explain the variability in ER difficulties across different groups.

The results of this study contribute significantly to research on ER difficulties in depression and anxiety disorders. They extend previous studies by examining the transdiagnostic nature of ER and RNT at the symptom subscale level in a clinical sample with diagnoses confirmed by the Structured Clinical Interview for DSM‐5. However, some limitations must be considered when interpreting the results of our study. First, we combined several anxiety‐related disorders in our anxiety group and mixed group (e.g., agoraphobia and/or panic disorder and/or PTSD). Although these disorders share similarities in their symptoms, they may differ in ER difficulties and RNT. For example, McEvoy et al. [25] found significantly more RNT in individuals with generalized anxiety disorder and social phobia compared to those with panic disorder. Additionally, the proportion of individuals with PTSD is higher in the mixed group than in the anxiety‐only group, which may further influence the results. Second, our diagnostic groups were based on the inclusion diagnoses of the overarching project, without considering other potential diagnoses. This may have reduced the diagnostic specificity of the depression and anxiety groups. Since not all diagnoses in the area of anxiety‐related disorders were included, there may have been individuals in our depression group with additional comorbid disorders, such as generalized anxiety disorder or social phobia. This could explain why we found no significant differences in anxiety symptoms between the anxiety and depression groups and why the differences between the depression group and the mixed anxiety and depression group were not as clear as those between the anxiety group and the mixed group. Third, due to different sample sizes across groups, our test procedures were less robust. Although we used nonparametric analyses to compensate, these analyses sometimes yielded different results, which may reflect the overly conservative nature of nonparametric methods. Fourth, the matching procedure for the nonclinical control group was matched to the overall clinical sample rather than to each diagnostic subgroup. This approach was chosen for methodological reasons, as subgroup‐specific matching would have required multiple control groups and a different analytic strategy. While differences in gender distribution may therefore represent a potential confounding factor, differences in employment status and physical activity are likely to reflect functional consequences of mental disorders rather than demographic confounds, and matching on these variables would risk overmatching. Future research should aim to address these limitations by considering more specific diagnostic categorizations, implementing subgroup‐specific matching procedures, and ensuring balanced sample sizes to enhance the reliability of analysis methods, even if some assumptions are violated.

## 5. Conclusion

Our study highlights the transdiagnostic significance of ER difficulties and RNT in depression, anxiety‐related disorders, and their comorbid presentations. These findings contribute to the growing body of evidence indicating that ER difficulties and RNT are common features across mental disorders (e.g., [3, 79]), underscoring their potential as shared therapeutic targets.

Whereas transdiagnostic mechanisms were most prominent, we also observed preliminary evidence of disorder‐specific patterns, particularly in depression. Additionally, comorbid conditions appeared to exacerbate certain ER difficulties, aligning with prior findings that comorbidity is associated with more severe impairment (e.g., [25, 80, 81]). However, these patterns were less consistent and robust than the transdiagnostic evidence. Together with earlier research (e.g., [4, 20]), our findings suggest a complex interplay between shared and disorder‐specific factors in ER. To better understand these relationships, larger and more diverse samples—including a wider range of (comorbid) mental disorders—are needed. Specifically, future research should aim to clarify how comorbidity is linked to the severity and presentation of ER difficulties and whether these differences are specific to certain disorders.

Although the present study did not examine direct associations between ER difficulties and RNT, it is worth noting that several studies propose associations between these processes. For example, reduced emotional awareness and clarity have been associated with maladaptive forms of rumination, particularly abstract–analytic rumination [26, 27]. Similarly, nonacceptance of emotional experiences has been discussed as being conceptually linked to avoidance‐oriented regulation, with RNT representing one possible cognitive strategy for managing distress [29–31]. In line with these accounts, our sensitivity analyses suggested substantial overlap between ER difficulties and RNT, particularly in individuals with comorbid conditions, while still indicating robust elevations of RNT across all clinical groups.

Future research should directly examine whether specific ER difficulties may function as mediators between RNT and psychopathology or vice versa. Such analyses would help clarify whether ER deficits represent mechanisms through which RNT contributes to symptom maintenance or whether ER difficulties and RNT reflect parallel manifestations of broader emotional dysregulation. Such process‐oriented questions require longitudinal or experimental approaches. At the same time, the causal and temporal relationships between ER difficulties, RNT, and psychopathology remain uncertain. Clarifying whether these factors primarily act as risk factors, maintaining factors, or both, potentially forming a vicious cycle that perpetuates symptoms, underscores the need for longitudinal and process‐oriented studies. This distinction is crucial, as it can not only deepen our understanding of ER and RNT, but also inform tailored interventions. For instance, if certain ER difficulties are identified as risk factors, early intervention programs could be developed to prevent the onset of mental disorders. Conversely, if these difficulties are epiphenomena, resulting from the disorders themselves, treatments could focus on managing these symptoms to improve overall functioning.

## Author Contributions

**Eva Herzog**: conceptualization, data curation, writing – original draft. **Sebastian Wolf**: funding acquisition, supervision, project administration. **Thomas Studnitz, Britta Seiffer, Jana Welkerling, and Johanna-Marie Zeibig**: investigation. **Anna Katharina Frei and Celina L. Müller**: investigation, writing – review and editing. **Gorden Sudeck**: resources, writing – review and editing. **Mia Maria Günak and Tristan T. Nakagawa**: data curation, writing – review and editing. **Thomas Ehring**: supervision, writing – review and editing. **Leonie Sundmacher, Anna Lena Flagmeier, Lena Zwanzleitner, and Ander Ramos-Murguialday**: resources. **Stefan Peters**: resources, writing – review and editing. **Keisuke Takano**: formal analysis, validation, writing – review and editing. **Barbara Cludius**: conceptualization, writing – review and editing.

## Funding

The parent study was fully funded by the German Innovation Fund of the Federal Joint Committee (Grant 01NVF19022). Open Access funding enabled and organized by Projekt DEAL.

## Conflicts of Interest

Stefan Peters declares that the German Association for Health–Enhancing Physical Activity and Exercise Therapy maintains a training programme for psychiatry, psychosomatics, and addiction. All other authors declare no competing interests.

## Endnotes

1

2

3

## Supporting Information

Additional supporting information can be found online in the Supporting Information section.

## Supporting information


**Supporting Information** The supporting materials include additional analyses that provide further insights into the analyses presented in the main text. Table S1 and S2 detail the pairwise comparisons of demographic (S1) and clinical characteristic (S2) among subgroups. Table S3 and Table S4 present the results of semiparametric MANOVA, Scheirer‐Ray‐Hare test, and Dunn’s post‐hoc test for ER difficulties and RNT across subgroups (S3) and number of diagnoses (S4). Table S5 and Table S6 provide the outcomes of parametric post‐hoc comparisons, whereas Table S7 and Table S8 provide the nonparametric results of the same comparisons. Table S9 and Table S10 present the results of the sensitivity analysis, including the ANCOVA model examining the effects of depression and anxiety on RNT while controlling for overall ER difficulties (S9), as well as the corresponding post‐hoc comparisons (S10).

## Data Availability

The data supporting the findings of this study are publicly accessible on the Open Science Framework at https://osf.io/5rcuz/.

## Footnotes

1
^1^This was a relevant criterion for the ImPuls study [32].

2
^2^The assessments conducted in the parent study were intensive and involved multiple phases, including wearing accelerometer‐based sensors to monitor participants’ activity patterns. Additionally, the final assessment took place 12 months after the initial one, requiring a considerable commitment of time and effort from the participants.

3
^3^In the original preregistration of our study, we planned to use the Bonferroni correction to adjust *p*‐values for multiple testing. However, after a thorough review of the literature and methodological considerations, we decided to use the Bonferroni–Holm correction instead to ensure a better balance between controlling Type I errors and maintaining statistical power (e.g., [82]).
