# Supplementary material for: The Transdiagnostic Role of Emotion Regulation Difficulties and Repetitive Negative Thinking in Depression, Anxiety, and Their Comorbidity
Source: Depress Anxiety. 2026 May 4;2026:9949037. doi: 10.1155/da/9949037 (PMC13139714; doi:10.1155/da/9949037)
Supplement: Supplementary file 1 — Supporting Information The supporting materials include additional analyses that provide further insights into the analyses presented in the main text. Table S1 and S2 detail the pairwise comparisons of demographic (S1) and clinical characteristic (S2) among subgroups. Table S3 and Table S4 present the results of semiparametric MANOVA, Scheirer‐Ray‐Hare test, and Dunn’s post‐hoc test for ER difficulties and RNT across subgroups (S3) and number of diagnoses (S4). Table S5 and Table S6 provide the outcomes of parametric post‐hoc comparisons, whereas Table S7 and Table S8 provide the nonparametric results of the same comparisons. Table S9 and Table S10 present the results of the sensitivity analysis, including the ANCOVA model examining the effects of depression and anxiety on RNT while controlling for overall ER difficulties (S9), as well as the corresponding post‐hoc comparisons (S10). [file DA-2026-9949037-s001.docx]

**Table S1**

*Comparison of Demographic Characteristics between Subgroups (Chi-squared tests)*

| Variables | Categories | Control Group  vs.  Depression | | Control Group  vs.  Anxiety  Disorders | | Control Group  vs.  Depression & Anxiety Disorders | | Depression  vs.  Anxiety Disorders | | Depression  vs.  Depression & Anxiety Disorders | | Anxiety Disorders  vs.  Depression & Anxiety Disorders | |
| --- | --- | --- | --- | --- | --- | --- | --- | --- | --- | --- | --- | --- | --- |
| Gender |  | **χ^2^ = 6.86(2),  *p* = .032** | | **χ^2^ = 24.42(2),  *p* < .001** | | **χ^2^ = 15.47(2),  *p* < .001** | | **χ^2^ = 7.78(2),  *p* = .020** | | **χ^2^ = 7.85(2),  *p* = .020** | | χ^2^ = 1.40(2),  *p* = .496 | |
|  |  | CG | D | CG | A | CG | D&A | D | A | D | D&A | A | D&A |
|  | Women | 1.64 | -1.64 | -0.20 | 0.20 | -1.56 | 1.56 | -1.91 | 1.91 | **-2.40** | **2.40** | - | |
|  | Men | -1.29 | 1.29 | 1.24 | -1.24 | **2.09** | **-2.09** | 1.14 | -1.14 | **2.71** | **-2.71** | - | |
|  | Other | **-2.26** | **2.26** | **-4.84** | **4.84** | **-3.39** | **3.39** | **2.22** | **-2.22** | -0.92 | 0.92 | - | |
| Employment |  | **χ^2^ = 22.84(2),  *p* < .001** | | **χ^2^ = 16.85(2),  *p* < .001** | | **χ^2^ = 31.73(2),  *p* < .001** | | χ^2^ = 0.67(2),  *p* = .716 | | χ^2^ = 5.85(2),  *p* = .054 | | χ^2^ = 2.20(2),  *p* = .333 | |
|  |  | CG | D | CG | A | CG | D&A | D | A | D | D&A | A | D&A |
|  | Unemployed | **-4.45** | **4.45** | **-3.93** | **3.93** | **-4.58** | **4.58** | - | | - | | - | |
|  | Employed | **4.63** | **-4.63** | **3.88** | **-3.88** | **5.63** | **-5.63** | - | | - | | - | |
|  | Other | -1.02 | 1.02 | -0.62 | 0.62 | **-2.54** | **2.54** | - | | - | | - | |
| No. of diagnoses^a^ |  | **G = 791.80(3),  *p* < .001** | | **G = 369.13(3),  *p* < .001** | | **G = 372.96(3),  *p* = .001** | | G = 0.49(3),  *p* = .921 | | **G = 102.66(3),  *p* < .001** | | **G = 66.94(3),  *p* <.001** | |
|  |  | CG | D | CG | A | CG | D&A | D | A | D | D&A | A | D&A |
|  | 0 | **9.10** | **-11.84** | **3.09** | **-7.45** | **3.14** | **-7.49** | - | | - | | - | |
|  | 1 | **-9.04** | **11.75** | **-5.54** | **13.35** | - | | - | | **2.96** | **-5.49** | **4.29** | **-4.26** |
|  | 2 | **-6.29** | **8.18** | **-3.92** | **9.44** | **-5.05** | **12.08** | - | | **-1.03** | **1.90** | **-1.19** | **1.18** |
|  | 3+ | **-4.34** | **5.65** | **-3.06** | **7.38** | **-5.54** | **13.23** | - | | **-2.93** | **5.39** | **-2.55** | **2.53** |
| Previous outpatient treatment |  | **χ^2^ = 175.28(1),  *p* < .001** | | **χ^2^ = 174.77(1),  *p* < .001** | | **χ^2^ = 126.39(1),  *p* < .001** | | **χ^2^ = 14.58(1),  *p* < .001** | | χ^2^ = 2.68(1),  *p* = .102 | | **χ^2^ = 4.44(1),  *p* < .001** | |
| Previous inpatient treatment |  | **χ^2^ = 177.68(1),  *p* < .001** | | **χ^2^ = 173.52(1),  *p* < .001** | | **χ^2^ = 177.00(1),  *p* < .001** | | χ^2^ = 2.40(1),  *p* = .121 | | χ^2^ = 2.72(1),  *p* = .099 | | χ^2^ = 0.01(1),  *p* = .942 | |
| Current psychological treatment |  | **χ^2^ = 312.80(1),  *p <* .001** | | **χ^2^ = 312.71(1),  *p* < .001** | | **χ^2^ = 296.69(1),  *p* < .001** | | χ^2^ = 1.46(1),  *p =* .227 | | χ^2^ = 0.55(1),  *p* = .459 | | χ^2^ = 0.15(1),  *p* = .704 | |
| Current pharmacological treatment |  | **χ^2^ = 240.56(1),  *p* < .001** | | **χ^2^ = 138.26(1),  *p* < .001** | | **χ^2^ = 180.38(1),  *p* < .001** | | χ^2^ = 1.94(1),  *p* = .164 | | χ^2^ < 0.01(1),  *p* = .987 | | χ^2^ = 1.28(1),  *p* = .257 | |

*Note.* For comparisons of variables with more than two categories, standardized residuals are reported to indicate where significant differences occur; residuals > |2| are considered significant; significant results are highlighted in bold; CG = Non-Clinical Control Group; A = Anxiety; D = Depression; D&A = Depression and Anxiety;

^a^ G-tests were used instead of chi-squared tests

**Table S2**

*Comparison of Clinical Characteristics between Subgroups (Dunn’s tests)*

| Variables | Control Group  vs.  Depression | | Control Group  vs.  Anxiety  Disorders | | Control Group  vs.  Depression & Anxiety Disorders | | Depression  vs.  Anxiety Disorders | | Depression  vs.  Depression & Anxiety Disorders | | Anxiety Disorders  vs.  Depression & Anxiety Disorders | |
| --- | --- | --- | --- | --- | --- | --- | --- | --- | --- | --- | --- | --- |
|  | *z* | *p* | *z* | *p* | *z* | *p* | *z* | *p* | *z* | *p* | *z* | *p* |
| Physical activity (minutes/week),  self-report (BSA-F) | **4.83** | **<.001** | 0.40 | .686 | 1.42 | .157 | **-2.51** | **.012** | -1.55 | .121 | 0.77 | .440 |
| Global symptom severity (BSI-18) | **-18.92** | **<.001** | **-10.91** | **<.001** | **-14.52** | **<.001** | 0.94 | .347 | **-2.42** | **.016** | **-2.70** | **.007** |
| Sleep quality  (PSQI) | **-14.91** | **<.001** | **-7.61** | **<.001** | **-11.97** | **<.001** | 1.51 | .130 | **-2.51** | **.012** | **-3.22** | **.001** |
| Perceived stress  (PSS) | **-18.91** | **<.001** | **-8.93** | **<.001** | **-13.15** | **<.001** | **2.88** | **.004** | -1.06 | .290 | **-3.18** | **.001** |
| Depressive symptoms  (PHQ-9) | **-19.77** | **<.001** | **-8.81** | **<.001** | **-14.06** | **<.001** | **3.38** | **<.001** | -1.47 | .142 | **-3.91** | **<.001** |
| Anxiety aymptoms (GAD-7) | **-18.19** | **<.001** | **-11.30** | **<.001** | **-13.95** | **<.001** | 0.13 | .893 | **-2.39** | **.017** | **-2.03** | **.043** |
| PTSD symptoms (PCL-5) | **-17.94** | **<.001** | **-10.58** | **<.001** | **-15.20** | **<.001** | 0.69 | .493 | **-3.71** | **<.001** | **-3.53** | **<.001** |
| Emotion regulation difficulties (DERS) | **-18.02** | **<.001** | **-9.45** | **<.001** | **-13.01** | **<.001** | 1.85 | .065 | -1.47 | .141 | **-2.68** | **.007** |
| Positive affect (PANAS) | **17.26** | **<.001** | **7.48** | **<.001** | **12.14** | **<.001** | **-3.26** | **.001** | 1.20 | .273 | **3.52** | **.004** |
| Negative affect (PANAS) | **-18.12** | **<.001** | **-10.44** | **<.001** | **-12.84** | **<.001** | 0.85 | .393 | -1.36 | .174 | -1.78 | .076 |

*Note.* BSI-18 = Brief Symptom Inventory; PSQI = Pittsburgh Sleep Quality Index; PSS = Perceived Stress Scale; PHQ-9 = Patient Health Questionnaire-9; GAD-7 = Generalized Anxiety Disorder Scale; PCL-5 = PTSD Checklist for DSM-5; PANAS = Positive and Negative Affect Schedule;

**Table S3**

*Means, Standard Deviations, and results of semi-parametric MANOVA, Scheirer-Ray-Hare test and post-hoc Dunn’s test for Emotion Regulation Difficulties Subscales and Repetitive Negative Thinking - Comparison of the Subgroups*

| Variable | Group | | | |  |  |
| --- | --- | --- | --- | --- | --- | --- |
|  | Control Group  *M* (*SD*) | Depression  *M* (*SD*) | Anxiety Disorders  *M* (*SD*) | Depression &  Anxiety Disorders  *M* (*SD*) | Test of between-subject effects/Scheirer-Ray-Hare | Significant differences in post-hoc comparisons |
| DERS: Nonacceptance | 10.15 (4.12)^a^ | 17.95 (5.66)^c^ | 16.62 (6.98)^e^ | 19.85 (5.62)^f^ | *H*_(1, 722)_=16.44^***^ | D&A > A > CG; D > CG |
| DERS: Goals | 10.25 (3.78)^a^ | 17.45 (4.04)^c^ | 16.09 (4.83)^e^ | 19.23 (4.31)^f^ | *H*_(1, 722)_=18.31^***^ | D&A > A > CG; D > CG |
| DERS: Impulse | 9.12 (3.17)^a^ | 14.32 (4.92)^c^ | 14.49 (5.86)^e^ | 17.09 (6.11)^f^ | *H*_(1, 722)_=13.45^***^ | D&A, D, A > CG |
| DERS: Awareness | 14.49 (4.56)^a^ | 19.88 (4.60)^c^ | 18.38 (4.81)^e^ | 19.23 (5.36)^f^ | *H*_(1, 722)_=19.87^***^ | D&A, D, A > CG |
| DERS: Strategies | 12.39 (4.29)^a^ | 25.57 (6.96)^c^ | 22.89 (7.45)^e^ | 27.80 (6.95)^f^ | *H*_(1, 722)_=30.64^***^ | D&A > A > CG; D > CG |
| DERS: Clarity | 8.15 (2.76)^a^ | 13.98 (4.57)^c^ | 12.69 (4.76)^e^ | 14.14 (4.78)^f^ | *H*_(1, 722)_=26.30^***^ | D&A, D, A > CG |
| PTQ | 14.81 (11.41)^b^ | 39.39 (10.53)^d^ | 37.26 (11.50)^e^ | 43.49 (9.75)^e^ | *H*_(1, 718)_=31.62^***^ | D&A, D, A > CG |

*Note.* DERS = Difficulties in Emotion Regulation Scale; PTQ = Perseverative Thinking Questionnaire; CG = Non-Clinical Control Group; D = Depression; A = Anxiety Disorders; D&A = Depression and Anxiety Disorders;

^a^ based on 375 participants; ^b^ based on 373 participants; ^c^ based on 220 participants; ^d^ based on 219 participants; ^e^ based on 65 participants; ^f^ based on 66 participants

*^*^p* < .05. ^**^*p* < .01. ^***^*p* < .001

**Table S4**

*Means, Standard Deviations, and results of MANOVA, ANOVAs and post-hoc t-tests for Emotion Regulation Difficulties Subscales and Repetitive Negative Thinking - Comparison of the number of diagnoses*

| Variable | Group | | | |  |  |
| --- | --- | --- | --- | --- | --- | --- |
|  | No diagnosis  *M* (*SD*) | 1 diagnosis  *M* (*SD*) | 2 diagnoses  *M* (*SD*) | 3+ diagnoses  *M* (*SD*) | Test of between-subject effects/Welch’s ANOVA | Significant differences in post-hoc comparisons |
| DERS: Nonacceptance | 10.15 (4.12) | 17.93 (5.66) | 17.27 (6.28) | 19.49 (6.06) | *F*_(3, 209)_=145.14^***^ | 3+, 2, 1, > 0 |
| DERS: Goals | 10.24 (3.78) | 17.13 (4.07) | 17.06 (4.71) | 19.11 (4.05) | *F*_(3, 224)_=204.47^***^ | 3+ > 2,1 > 0 |
| DERS: Impulse | 9.12 (3.17) | 13.96 (4.93) | 15.03 (5.84) | 16.65 (5.48) | *F*_(3, 202)_=104.04^**^ | 3+ > 1; 3+, 2 ,1 > 0 |
| DERS: Awareness | 14.49 (4.56) | 19.87 (4.73) | 18.95 (4.79) | 19.42 (5.00) | *F*_(3, 228)_=69.07^***^ | 3+, 2, 1 > 0 |
| DERS: Strategies | 12.39 4.27) | 25.18 (6.91) | 24.60 (7.74) | 27.47 (6.68) | *F*_(3, 205)_=303.36^***^ | 3+ > 2; 3+, 2, 1 > 0 |
| DERS: Clarity | 8.15 (2.76) | 13.99 (4.63) | 13.23 (4.78) | 14.08 (4.54) | *F*_(3, 203)_=128.74^***^ | 3+, 2, 1 > 0 |
| PTQ | 14.81 (11.41) | 38.87 (9.90) | 39.55 (12.03) | 41.96 (10.16) | χ^2^ (3) = 401.86^***h^ | 3+, 2, 1 > 0 |

*Note.* DERS = Difficulties in Emotion Regulation Scale; PTQ = Perseverative Thinking Questionnaire; 0 = no diagnosis; 1 = one diagnosis; 2 = two diagnoses; 3+ = three or more diagnoses

^a^ based on 375 participants; ^b^ based on 373 participants; ^c^ based on 165 participants; ^d^ based on 164 participants; ^e^ based on 110 participants; ^f^ based on 109 participants; ^g^ based on 76 participants; ^h^ Kruskal-Wallis test

*^*^p* < .05. ^**^*p* < .01. ^***^*p* < .001

**Table S5**

*Post-hoc Comparisons of Subgroups on Emotion Regulation and Repetitive Negative Thinking (t-tests)*

| Variable | Comparisons | | | | | | | | | | | | | | | | | |
| --- | --- | --- | --- | --- | --- | --- | --- | --- | --- | --- | --- | --- | --- | --- | --- | --- | --- | --- |
|  | Control Group  vs.  Depression | | | Control Group  vs.  Anxiety Disorders | | | Control Group  vs.  Depression & Anxiety Disorders | | | Depression  vs.  Anxiety Disorders | | | Depression  vs.  Depression & Anxiety Disorders | | | Anxiety Disorders  vs.  Depression & Anxiety Disorders | | |
|  | *t* | *df* | *p* | *t* | *df* | *p* | *t* | *df* | *p* | *t* | *df* | *p* | *t* | *df* | *p* | *t* | *df* | *p* |
| DERS: Nonacceptance | **-17.87** | **356.42** | **<.001** | **-7.25** | **71.91** | **<.001** | **-13.40** | **77.75** | **<.001** | 1.41 | 90.22 | .161 | **-2.40** | **107.52** | **.037** | **-2.92** | **122.60** | **.013** |
| DERS: Goals | **-21.51** | **433.92** | **<.001** | **-9.28** | **78.12** | **<.001** | **-15.89** | **83.47** | **<.001** | **2.07** | **92.07** | **.041** | **-2.97** | **101.73** | **.007** | **-3.92** | **126.90** | **<.001** |
| DERS: Impulse | **-14.06** | **327.13** | **<.001** | **-7.22** | **70.64** | **<.001** | **-10.37** | **71.29** | **<.001** | -0.21 | 92.36 | .832 | **-3.37** | **91.80** | **.003** | **-2.49** | **128.91** | **.028** |
| DERS: Awareness | **-13.85** | **85.12** | **<.001** | **-6.07** | **85.12** | **<.001** | **-6.76** | **82.38** | **<.001** | 2.23 | 100.97 | .085 | -0.90 | 95.43 | .692 | 0.95 | 127.91 | .692 |
| DERS: Strategies | **-25.40** | **317.96** | **<.001** | **-11.05** | **71.51** | **<.001** | **-17.44** | **73.93** | **<.001** | **2.59** | **99.36** | **.022** | **-2.29** | **107.15** | **.024** | **-3.90** | **128.08** | **<.001** |
| DERS: Clarity | **-17.17** | **314.71** | **<.001** | **-7.48** | **71.67** | **<.001** | **-9.89** | **72.83** | **.001** | 1.93 | 101.43 | .169 | -0.24 | 103.17 | .811 | -1.73 | 128.99 | .171 |
| PTQ | **-26.58** | **486.49** | **<.001** | **-14.55** | **87.41** | **<.001** | **-21.64** | **98.61** | **<.001** | 1.33 | 98.08 | .185 | **-2.97** | **114.00** | **.007** | **-3.36** | **123.91** | **.003** |

*Note.* DERS = Difficulties in Emotion Regulation Scale; PTQ = Perseverative Thinking Questionnaire; significant results are highlighted in bold

**Table S6**

*Post-hoc Analysis of Group Differences by Diagnosis Count (t-tests)*

| Variable | Comparisons | | | | | | | | | | | | | | | | | |
| --- | --- | --- | --- | --- | --- | --- | --- | --- | --- | --- | --- | --- | --- | --- | --- | --- | --- | --- |
|  | no diagnosis  vs.  one diagnosis | | | no diagnosis  vs.  two diagnoses | | | no diagnosis  vs.  three or more diagnoses | | | one diagnosis  vs.  two diagnoses | | | one diagnosis  vs.  three or more diagnoses | | | two diagnoses  vs.  three or more diagnoses | | |
|  | *t* | *df* | *p* | *t* | *df* | *p* | *t* | *df* | *p* | *t* | *df* | *p* | *t* | *df* | *p* | *t* | *df* | *p* |
| DERS: Nonacceptance | **-15.90** | **243.58** | **<.001** | **-11.21** | **137.66** | **<.001** | **-12.85** | **89.57** | **<.001** | 0.89 | 216.86 | .375 | -1.89 | 137.30 | .122 | -2.41 | 165.05 | .051 |
| DERS: Goals | **-18.50** | **293.03** | **<.001** | **-13.91** | **152.46** | **<.001** | **-17.57** | **103.07** | **<.001** | 0.14 | 210.23 | .886 | **-3.50** | **146.56** | **.002** | **-3.17** | **175.22** | **.004** |
| DERS: Impulse | **-11.60** | **225.88** | **<.001** | **-10.19** | **128.40** | **<.001** | **-11.59** | **85.43** | **<.001** | -1.58 | 202.27 | .115 | **-3.65** | **132.82** | **.001** | -1.93 | 167.71 | .111 |
| DERS: Awareness | **-12.30** | **303.44** | **<.001** | **-8.68** | **171.33** | **<.001** | **-7.95** | **101.84** | **<.001** | 1.57 | 231.87 | .351 | 0.65 | 138.74 | 1.00 | -0.65 | 156.71 | 1.00 |
| DERS: Strategies | **-22.01** | **221.52** | **<.001** | **-15.85** | **129.17** | **<.001** | **-18.90** | **87.90** | **<.001** | 0.64 | 215.07 | .525 | **-2.45** | **150.24** | **.031** | **-2.70** | **175.01** | **.023** |
| DERS: Clarity | **-15.07** | **217.22** | **<.001** | **-10.63** | **131.06** | **<.001** | **-10.98** | **86.58** | **<.001** | 1.31 | 228.41 | .576 | -0.14 | 148.30 | .886 | -.123 | 166.59 | .576 |
| PTQ | **-24.73** | **355.69** | **<.001** | **-19.12** | **168.90** | **<.001** | **-20.78** | **116.94** | **<.001** | -0.49 | 200.33 | .625 | -2.21 | 142.84 | .086 | -1.47 | 176.28 | .286 |

*Note.* DERS = Difficulties in Emotion Regulation Scale; PTQ = Perseverative Thinking Questionnaire; significant results are highlighted in bold

**Table S7**

*Nonparametric Post-hoc Comparisons of Subgroups on Emotion Regulation and Repetitive Negative Thinking (Dunn’s Test)*

| Variable | Comparisons | | | | | | | | | | | |
| --- | --- | --- | --- | --- | --- | --- | --- | --- | --- | --- | --- | --- |
|  | Control Group  vs.  Depression | | Control Group  vs.  Anxiety  Disorders | | Control Group  vs.  Depression & Anxiety Disorders | | Depression  vs.  Anxiety Disorders | | Depression  vs.  Depression & Anxiety Disorders | | Anxiety Disorders  vs.  Depression & Anxiety Disorders | |
|  | *z* | *p* | *z* | *p* | *z* | *p* | *z* | *p* | *z* | *p* | *z* | *p* |
| DERS:Nonacceptance | **-15.04** | **<.001** | **-7.56** | **<.001** | **-11.27** | **<.001** | 1.85 | .128 | -1.62 | .105 | **-2.80** | **.015** |
| DERS:Goals | **-15.86** | **<.001** | **-8.19** | **<.001** | **-12.10** | **<.001** | 1.75 | .081 | -1.91 | .111 | **-2.95** | **.009** |
| DERS:Impulse | **-12.92** | **<.001** | **7.78** | **<.001** | **-10.71** | **<.001** | 0.37 | .714 | -2.37 | .053 | -2.20 | .056 |
| DERS:Awareness | **-12.06** | **<.001** | **-5.34** | **<.001** | **-6.56** | **<.001** | 2.17 | .091 | 1.05 | .584 | -0.90 | .366 |
| DERS:Strategies | **-18.08** | **<.001** | **-9.36** | **<.001** | **-12.87** | **<.001** | 1.96 | .099 | -1.30 | .194 | **-2.63** | **.026** |
| DERS:Clarity | **-14.87** | **<.001** | **-7.47** | **<.001** | **-9.51** | **<.001** | 1.84 | .199 | 0.05 | .960 | -1.52 | .255 |
| PTQ | **-17.21** | **<.001** | **-10.02** | **<.001** | **-12.76** | **<.001** | 0.84 | .399 | -1.77 | .154 | -2.10 | .106 |

*Note.* DERS = Difficulties in Emotion Regulation Scale; PTQ = Perseverative Thinking Questionnaire; significant results are highlighted in bold

**Table S8**

*Nonparametric Post-hoc Comparisons of the Number of Diagnoses*

| Variable | Comparisons | | | | | | | | | | | | | | | | | | | | | | | |
| --- | --- | --- | --- | --- | --- | --- | --- | --- | --- | --- | --- | --- | --- | --- | --- | --- | --- | --- | --- | --- | --- | --- | --- | --- |
|  | no diagnosis  vs.  one diagnosis | | | | no diagnosis  vs.  two diagnoses | | | | no diagnosis  vs.  three or more diagnoses | | | | one diagnosis  vs.  two diagnoses | | | | one diagnosis  vs.  three or more diagnoses | | | | two diagnoses  vs.  three or more diagnoses | | | |
|  | *M* | 95% CI | | *p* | *M* | 95% CI | | *p* | *M* | 95% CI | | *p* | *M* | 95% CI | | *p* | *M* | 95% CI | | *p* | *M* | 95% CI | | *p* |
| DERS: Nonacceptance | **7.78** | **[6.52, 9.05]** | | **<.001** | **7.12** | **[5.47, 8.78]** | | **<.001** | **9.34** | **[7.43, 11.24]** | | **<.001** | -0.66 | [-2.59, 1.26] | | .811 | 1.55 | [-0.59, 3.69] | | .238 | 2.21 | [-0.17, 4.59] | | .078 |
| DERS: Goals | **6.89** | **[5.92, 7.85]** | | **<.001** | **6.81** | **[5.54, 8.08]** | | **<.001** | **8.86** | **[7.54, 10.17]** | | **<.001** | -0.08 | [-1.50, 1.34] | | .999 | **1.97** | **[0.51, 3.43]** | | **.003** | **2.05** | **[0.38, 3.73]** | | **.010** |
| DERS: Impulse | **4.84** | **[3.76, 5.92]** | | **<.001** | **5.91** | **[4.40, 7.42]** | | **<.001** | **7.53** | **[5.82, 9.23]** | | **<.001** | 1.07 | [-0.68, 2.82] | | .391 | **2.69** | **[0.77, 4.60]** | | **.002** | 1.62 | [-0.56, 3.80] | | .221 |
| DERS: Awareness | **5.38** | **[4.25, 6.51]** | | **<.001** | **4.45** | **[3.12, 5.79]** | | **<.001** | **4.93** | **[3.31, 6.55]** | | **<.001** | -0.92 | [-2.44, 0.60] | | .397 | -0.45 | [-2.22, 1.33] | | .914 | 0.48 | [-1.43, 2.38] | | .916 |
| DERS: Strategies | **12.79** | **[11.29, 14.30]** | | **<.001** | **12.21** | **[10.21, 14.22]** | | **<.001** | **15.08** | **[12.99, 17.17]** | | **<.001** | -0.58 | [-2.95, 1.78] | | .920 | 2.29 | [-0.14, 4.72] | | .073 | **2.87** | **[0.11, 5.63]** | | **.038** |
| DERS: Clarity | **5.84** | **[4.84, 6.84]** | | **<.001** | **5.08** | **[3.83, 6.32]** | | **<.001** | **5.93** | **[4.51, 7.34]** | | **<.001** | -0.76 | [-2.26, 0.74] | | .558 | 0.09 | [-1.55, 1.74] | | .999 | 0.85 | [-0.94, 2.65] | | .609 |
|  | *z* | | *p* | | *z* | | *p* | | *z* | | *p* | | *z* | | *p* | | *z* | | *p* | | *z* | | *p* | |
| PTQ | **-15.41** | | **<.001** | | **-13.46** | | **<.001** | | **-12.90** | | **<.001** | | -0.18 | | .855 | | -1.29 | | .586 | | -1.05 | | .587 | |

*Note.* DERS = Difficulties in Emotion Regulation Scale; PTQ = Perseverative Thinking Questionnaire; significant results are highlighted in bold

**Table S9**

*Sensitivity analysis: ANCOVA predicting repetitive negative thinking (PTQ) with overall emotion regulation difficulties (DERS) as covariate*

| Effect | *df* | *F* | *p* |
| --- | --- | --- | --- |
| Anxiety (presence vs. absence) | 1, 717 | 58.96 | <.001 |
| Depression (presence vs. absence) | 1, 717 | 84.41 | <.001 |
| Anxiety x Depression | 1, 717 | 25.88 | <.001 |
| ER difficulties (DERS total) | 1, 717 | 363.23 | <.001 |

*Note.* PTQ = Perseverative Thinking Questionnaire; DERS = Difficulties in Emotion Regulation Scale

**Table S10**

*Pairwise comparisons of repetitive negative thinking (PTQ) between diagnostic groups adjusted for overall emotion regulation difficulties (DERS)*

| Comparison | Mean difference | *SE* | *t* | *P (Holm)* |
| --- | --- | --- | --- | --- |
| Control Group vs. Anxiety Disorders | -10.45 | 1.36 | -7.68 | <.001 |
| Control Group vs. Depression | -9.95 | 1.08 | -9.19 | <.001 |
| Control Group vs. Depression & Anxiety Disorders | -11.17 | 1.52 | -7.37 | <.001 |
| Anxiety Disorders vs. Depression | 0.50 | 1.27 | 0.39 | 1.000 |
| Anxiety Disorders vs. Depression & Anxiety Disorders | -0.72 | 1.60 | -0.45 | 1.000 |
| Depression vs. Depression & Anxiety Disorders | -1.22 | 1.28 | -0.96 | 1.000 |

*Note.* PTQ = Perseverative Thinking Questionnaire; DERS = Difficulties in Emotion Regulation Scale; Values represent differences in estimated marginal means adjusted for overall ER difficulties (DERS total score). Positive values indicate higher PTQ scores in the second group. *p* values are Holm-corrected for multiple comparisons.
